# Efficacy, safety and tolerability of ongoing statin plus ezetimibe versus doubling the ongoing statin dose in hypercholesterolemic Taiwanese patients: an open-label, randomized clinical trial

**DOI:** 10.1186/1756-0500-5-251

**Published:** 2012-05-23

**Authors:** Chih-Chieh Yu, Wen-Ter Lai, Kuang-Chung Shih, Tsung-Hsien Lin, Chieh-Hua Lu, Hung-Jen Lai, Mary E Hanson, Juey-Jen Hwang

**Affiliations:** 1 National Taiwan University Hospital, Taipei, Taiwan; 2 Division of Cardiology, Department of Internal Medicine, Kaohsiung Medical University Hospital and Department of Internal Medicine, Faculty of Medicine, College of Medicine, Kaohsiung Medical University, Kaohsiung, Taiwan; 3 Tri-Service General Hospital, Taipei, Taiwan; 4 MSD Taiwan, Taipei, Taiwan; 5 Merck Sharp & Dohme Corp, Whitehouse Station, NJ, USA; 6 Cardiovascular Division, Department of Internal Medicine, National Taiwan University College of Medicine and Hospital, Taipei, Taiwan

**Keywords:** Ezetimibe, Simvastatin, Atorvastatin, Pravastatin

## Abstract

**Background:**

Reducing low-density lipoprotein cholesterol (LDL-C) is associated with reduced risk for major coronary events. Despite statin efficacy, a considerable proportion of statin-treated hypercholesterolemic patients fail to reach therapeutic LDL-C targets as defined by guidelines. This study compared the efficacy of ezetimibe added to ongoing statins with doubling the dose of ongoing statin in a population of Taiwanese patients with hypercholesterolemia.

**Methods:**

This was a randomized, open-label, parallel-group comparison study of ezetimibe 10 mg added to ongoing statin compared with doubling the dose of ongoing statin. Adult Taiwanese hypercholesterolemic patients not at optimal LDL-C levels with previous statin treatment were randomized (N = 83) to ongoing statin + ezetimibe (simvastatin, atorvastatin or pravastatin + ezetimibe at doses of 20/10, 10/10 or 20/10 mg) or doubling the dose of ongoing statin (simvastatin 40 mg, atorvastatin 20 mg or pravastatin 40 mg) for 8 weeks. Percent change in total cholesterol, LDL-C, high-density lipoprotein cholesterol (HDL-C) and triglycerides, and specified safety parameters were assessed at 4 and 8 weeks.

**Results:**

At 8 weeks, patients treated with statin + ezetimibe experienced significantly greater reductions compared with doubling the statin dose in LDL-C (26.2% vs 17.9%, p = 0.0026) and total cholesterol (20.8% vs 12.2%, p = 0.0003). Percentage of patients achieving treatment goal was greater for statin + ezetimibe (58.6%) vs doubling statin (41.2%), but the difference was not statistically significant (p = 0.1675). The safety and tolerability profiles were similar between treatments.

**Conclusion:**

Ezetimibe added to ongoing statin therapy resulted in significantly greater lipid-lowering compared with doubling the dose of statin in Taiwanese patients with hypercholesterolemia. Studies to assess clinical outcome benefit are ongoing.

**Trial registration:**

Registered at ClinicalTrials.gov: NCT00652327

## Background

Chinese populations are perceived to have lower low-density lipoprotein cholesterol (LDL-C) levels and coronary heart disease (CHD) risk than Caucasians [1]. However, as life expectancy and the standard of living improve in Asian countries, an increased risk for cardiovascular disease, high blood pressure, diabetes and dyslipidemia have become more prevalent and are predicted to continue to rise as the population ages [2]. LDL-C is the primary target of cholesterol-lowering therapy. Reducing serum levels of LDL-C is associated with a reduction in risk for major coronary events [3-9] and greater reduction in ischemic heart disease [10].

The National Cholesterol Education Program Adult Treatment Panel III (NCEP ATP III), American Heart Association/American College of Cardiology (AHA/ACC) and European Society of Cardiology (ESC) guidelines recommend therapeutic life changes (TLCs) as the first line of treatment; and in patients who do not meet lipid-lowering targets with TLCs, addition of medication may be recommended [11-13]. HMG-CoA reductase inhibitors (statins) are the first line of therapy as an adjunct to TLCs [14,15]. They inhibit cholesterol synthesis in the liver and dose-dependent reductions in LDL-C range from 20% to 60%, depending on the drug and dosage used [16-21].

Despite statin efficacy, a considerable proportion of statin-treated patients with hypercholesterolemia fail to reach therapeutic LDL-C targets as defined by guidelines. This may be attributed to lack of use of adequate doses of statins, the extent of cholesterol lowering required in some individuals, or in some cases, safety and tolerability issues with high-dose statins. Moreover, health insurance regulations in Taiwan may play a role in prescribing practices that impact dosing and brand of statin [22,23]. Ezetimibe selectively inhibits the intestinal absorption of cholesterol and related phytosterols. Its molecular target is the sterol transporter, Niemann-Pick C1-like 1 (NPC1L1), which is responsible for cholesterol absorption in the intestine [24-30]. It has a mechanism of action that is complementary to that of statins and has been shown to reduce LDL-C levels significantly more than placebo (reviewed in [31]). Ezetimibe also improves other lipids such as high-density lipoprotein cholesterol (HDL-C), triglycerides, and Apo B (reviewed in [31]). Statin plus ezetimibe vs comparable doses of statin monotherapy show that coadministration is more effective than statin monotherapy at reducing LDL-C, even at high statin doses (reviewed in [31]). Ezetimibe has been shown to inhibit the development of atherosclerosis in Apo E knockout mice [32], and trials to assess clinical outcome benefit with ezetimibe treatment in humans are ongoing.

The primary objective of this study was to compare the LDL-C lowering efficacy of ezetimibe 10 mg added to ongoing statins (simvastatin 20 mg, atorvastatin 10 mg or pravastatin 20 mg) with doubling the dose of ongoing statin after 8 weeks of treatment in a population of Taiwanese patients with hypercholesterolemia. Secondary objectives were to compare the effect of ezetimibe 10 mg added to ongoing statins with doubling the dose of ongoing statin with respect to effects on total cholesterol, triglycerides and HDL-C; proportion of patients achieving therapeutic LDL-C targets (based on individual risk), safety and tolerability.

## Methods

### Study design and ethics

This was a randomized, open-label, parallel-group comparison study conducted at 3 sites in Taiwan between February 2006 and July 2007. The study was conducted according to the Declaration of Helsinki and the laws/regulations of the local health authorities as well as Good Clinical Practice guidelines. The study protocol and informed consent form were reviewed and approved by the institutional review boards of the three participating study sites (National Taiwan University Hospital-Yun Lin Branch, Tri-Service General Hospital, and Chung-Ho Memorial Hospital, Kaohsiung Medical University) and all patients gave written informed consent prior to commencement of any trial-related activities. At screening patients were instructed to follow the NCEP ATP III therapeutic lifestyle changes or similar cholesterol-lowering diet throughout the study.

### Patients

Men and women 18–80 years old with hypercholesterolemia who were unable to achieve NCEP ATP III recommended LDL-C treatment targets while taking current statin treatment (simvastatin 20 mg, atorvastatin 10 mg or pravastatin 20 mg alone for at least 12 weeks) were enrolled in the study. Inclusion criteria were triglycerides ≤400 mg/dL, liver transaminases (alanine aminotransferase [ALT] and aspartate aminotransferase [AST)]) ≤2 X ULN with no active liver disease, and creatine kinase (CK) level ≤2 X ULN. Women were required to use medically acceptable birth control and were excluded if they were pregnant or lactating. Patients were excluded if they had a history of mental illness, drug or alcohol abuse, treatment with other investigational drugs within 3 months of screening, active liver disease or renal impairment; unstable angina, uncontrolled cardiac arrhythmia, hypertension, diabetes, endocrine or metabolic disorders; or any condition or situation, which in the opinion of the investigator, might pose a risk to the patient or confound the results of the study. In addition, patients were excluded if they were taking any lipid-lowering agents other than the statins listed above, including fish oils, cholestin, bile-acid sequestrants, or niacin (>200 mg/d) within 6 weeks, or fibrates within 8 weeks of screening, or consuming >250 mL of grapefruit juice per day. Use of prescription and/or over-the counter-drugs with the potential for significant lipid effects (other than study drug), or with potential drug interactions with the statins were prohibited during the study.

### Treatments

Patients were randomized to 1 of 2 treatments: ongoing statin plus ezetimibe (simvastatin, atorvastatin or pravastatin plus ezetimibe at doses of 20/10, 10/10 or 20/10 mg/mg) or doubling the dose of ongoing statin (simvastatin 40 mg, atorvastatin 20 mg or pravastatin 40 mg) according to their ongoing statin use. All medications were dispensed by the study site pharmacies. Since this was an open-label study, there was no blinding and no need for identity of investigational product. Compliance was assessed at each follow-up visit by tablet counts.

### Efficacy endpoints

The primary efficacy endpoint was percent change from baseline in LDL-C after 8 weeks of treatment. Secondary efficacy endpoints were the percent change from baseline in total cholesterol, LDL-C, HDL-C, and triglycerides at Week 4 and Week 8, and the proportion of patients achieving NCEP ATP III-recommended treatment targets for LDL-C (<160 mg/dL to <100 mg/dL based on risk stratification at baseline) [11]. Lipid assessments were performed at a central laboratory in the National Taiwan University Hospital Yun Lin branch. LDL-C was calculated using the Friedewald equation. Plasma cholesterol and triglycerides were determined using enzymatic methods. HDL-C was measured after precipitation of the apoprotein beta-containing lipoproteins (LDL and VLDL) in whole plasma by heparin manganese chloride.

### Safety/tolerability endpoints

Adverse events were coded into COSTART preferred terms and body systems. The occurrence of serious adverse events or potentially dangerous abnormalities in safety tests warranted patient withdrawal from medication. Laboratory tests that were performed at baseline and at Week 8 included complete blood count, thyroid-stimulating hormone (TSH), serum creatinine, total bilirubin, gamma-glutamyl transpeptidase (GGT), ALT, AST, CK and urine analysis. Laboratory tests that were performed at Week 4 included ALT, AST and CK. Consecutive elevations of ALT or AST >3 X ULN were considered reason for discontinuation from the study.

### Statistics

It was calculated that with approximately 80 subjects, 40 in each treatment group, there would be at least 85% power to detect approximately 10% difference in the percentage improvement of LDL, assuming a standard deviation of 15% from previous studies, alpha = 0.05, 2 sided. The intent-to-treat (ITT) population, defined as all randomized patients who took at least one dose of study medication and had at least one post-randomization measurement, was used for the efficacy analysis. The last observation carried forward method was employed to adjust for any dropouts or missing data. Data from the 3 study centers were pooled with no adjustment for study site. Due to the small sample size, none of the endpoints of interest (LDL-C, total cholesterol, HDL-C, and triglycerides) followed a normal distribution. Therefore, these data were log-transformed and non-parametric methods were used to analyze the data. The primary efficacy variables were analyzed using the Wilcoxon signed rank test to compute the within-group differences and the Wilcoxon rank sum test for between-group differences.

In subjects with available endpoint data, the percentage of patients reaching prespecified treatment levels for LDL-C between primary and secondary hypercholesterolemia and between two treatment groups were assessed using the Chi-square test. Median percent change from baseline in LDL-C was assessed in patients grouped by baseline diabetic status using descriptive statistics only since this was a post hoc analysis and the group sizes were small. All significance tests are two-tailed with α = 0.05. All patients who were randomized into treatment and took at least one dose of medication were included in the safety analysis. Nonparametric methods were used to assess between- and within-groups differences in safety parameters.

## Results

**Figure 1:**
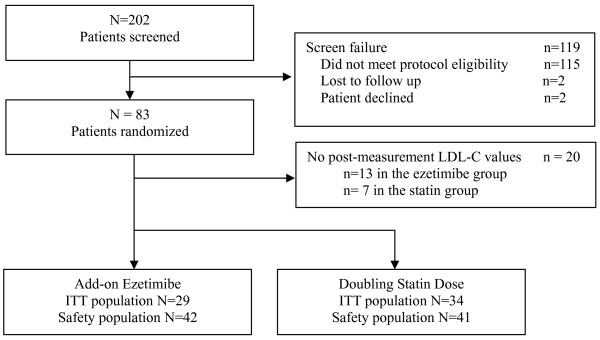
Dispositions of Patients.

The flow of participants through the study is shown in Figure 1. Of the 202 patients screened, 83 were randomized, 42 were assigned to the ezetimibe added to ongoing statin treatment group, 41 were assigned to the double statin dose treatment group, and 20 patients had no post-randomization LDL-C values (13 patients from the ezetimibe group and 7 patients from the statin group). Data from 5 subjects on statin treatment were missing from the study and included in the ITT analysis. Reasons for discontinuation included adverse event, 2 did not meet protocol eligibility, 1 subject did not wish to continue for reasons unrelated to study drug and 1 for "other" reasons. Baseline characteristics for each treatment group in the ITT population are summarized in Table 1. There were no statistically significant differences between the treatment groups in baseline demographics, with the exception of the mean age between the 2 groups, which was 55 years (SD = 10.9) in the ezetimibe group and 61 years (SD = 10.6) in the statin group (p = 0.0267).

**Table 1:** Summary of baseline characteristics of the ITT population

	**Ezetimibe** **(N = 29)**	**Statin** **(N = 34)**	**P-value**
Age, mean (SD)	54.2 (10.9)	61.2 (10.5)	0.0277*
Height, mean (SD)	164.0 (10.3)	159.5 (9.2)	0.5549*
Weight, mean (SD)	75.0 (15.7)	71.0 (11.2)	0.0733*
Sex			
Male, n (%)	17 (58.6)	18 (52.9)	0.7999^†^
Female, n (%)	12 (41.4)	16 (47.1)	---
Diabetes Mellitus, n (%)	14 (48.3)	16 (47.1)	0.9304*
Hypertension, n (%)	10 (34.5)	27 (79.4)	0.0007*
Coronary Artery Disease, n (%)	8 (27.6)	10 (29.4)	0.8814*
Total cholesterol, mean (SD)	213.6 (48.4)	207.3 (28.8)	0.8577*
LDL-C, mean (SD)	144.6 (45.8)	130.9 (19.4)	0.2119*
HDL-C, mean (SD)	48.4 (9.1)	52.5 (11.9)	0.1047*
Triglycerides, mean (SD)	140.3 (59.7)	158.5 (68.1)	0.3107*
ALT, mean (SD)	30.7 (12.6)	26.9 (8.1)	0.2381*
AST, mean (SD)	25.5 (7.7)	25.2 (7.8)	0.3761*
CK, mean (SD)	121. 5 (64.0)	108.0 (77.1)	0.1924*
GGT, mean (SD)	31.8 (16.5)^‡^	44.3 (32.0)	0.1318*

*ALT* Alanine aminotransferase; *AST* aspartate aminotransferase, *CK* creatine kinase, *GGT* gamma-glutamyl transpeptidase; *SD* standard deviation.

*Wilcoxon rank sum test.

^†^Chi-square test.

^‡^There was one subject in the ezetimibe group with no record for gamma-glutamyl transpeptidase.

At baseline in the ITT population, subjects in both treatment groups showed generally similar values on most of the laboratory tests results, except GGT and ALT. The GGT level of the ezetimibe group at baseline was 31.8, vs 44.3 in the statin group (p = 0.1318), and the ALT value of the ezetimibe group was numerically higher than that of the statin group (30.7 versus 26.9; p = 0.2381). Baseline lipid values were similar between groups; although LDL-C was numerically higher in the ezetimibe group vs the statin group and triglycerides were numerically higher in the statin group (Table 1).

**Figure 2:**
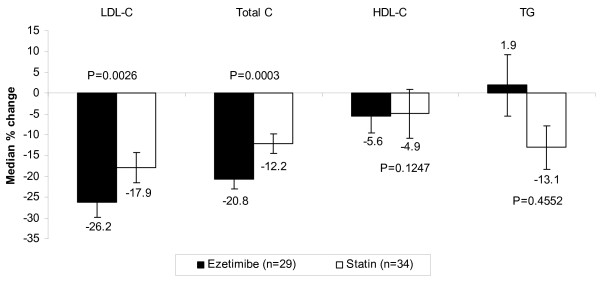
Percent change from baseline in lipid levels after 8 weeks of treatment (ITT population).

Median percent change from baseline and between-treatments difference at Week 8 in LDL-C and other lipids is shown in Figure 2. The addition of ezetimibe to ongoing statin therapy resulted in a significantly greater reduction from baseline in LDL-C levels compared with doubling the ongoing statin dose after 8 weeks of treatment (−26.2% vs −17.9%, p = 0.0026). The percent change from baseline for LDL-C at Week 4 was numerically higher for the ezetimibe group compared with the statin group, although the difference was not statistically significant (−21.9% vs −17.4%; p = 0.0645). There were significantly greater reductions with ezetimibe added to ongoing statin in total cholesterol at Week 4 (−19.2% vs −12.9%; p = 0.0068) and Week 8 (−20.8% vs −12.2%; p = 0.0003). There were no significant differences between treatments in the change from baseline in HDL-C at Week 4 (−3.7% vs −11.3%; p = 0.5371) or Week 8 (−5.6% vs 4.9%; p = 0.1274) nor triglycerides at Week 4 (1.8% vs 0.7%; p = 0.8529) or Week 8 (1.9% vs −13.1%; p = 0.4552). The number of patients reaching LDL-C treatment goals (determined by individual risk at randomization) after 8 weeks of treatment was greater in the combination treatment group, although there was no statistically significant difference between the treatment groups in the percent of patients attaining goals (p = 0.1675). A total of 17/29 (58.6%) patients taking ezetimibe added to ongoing statin attained LDL-C goal levels after 8 weeks of treatment and a total of 14/34 (41.2%) patients receiving double their baseline statin dose attained LDL-C goal levels after 8 weeks of treatment.

**Figure 3:**
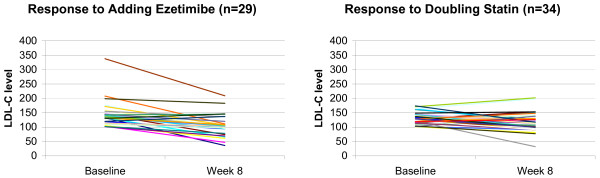
Inter-individual variability in response to treatment in Taiwanese patients with hypercholesterolemia.

Figure 3 shows the change from baseline in LDL-C levels in individual patients after 8 weeks of treatment. At baseline there was a wider range of LDL-C levels in patients who were randomized to the ezetimibe group (101 – 338 mg/dL) compared with those in the statin group (101 – 174 mg/dL). After 8 weeks, the range of LDL-C levels was nearly identical between the two treatment groups (36 – 209 mg/dL in the ezetimibe group compared with 32 – 202 mg/dL in the statin group). Among the statin-treated patients, 12 patients experienced an increase in LDL-C level, whereas 3 patients in the ezetimibe-treated group experienced an increase in LDL-C level.

In patients grouped by baseline diabetic status (yes/no), the median percent change from baseline in LDL-C was numerically greater in patients being treated with ezetimibe added to statin compared with patients who doubled their statin dose in both subgroups. In patients with diabetes, the median percent change ± standard deviation (SD) was −22.3% ±23.0 with the combination therapy vs. -16.1% ±29.5 with doubling the statin dose; and in patients without diabetes the median percent change ± SD was −30.1% ±17.0 with the combination treatment vs −7.2% ±20.8 with doubling the statin dose.

A summary of safety is shown in Table 2 for the entire randomized population. There was a total of 34 adverse events, with 18 events in the ezetimibe group and 16 in the statin group. A detailed list of adverse events and serious adverse events in the subset of patients who experienced adverse events is shown in Table 3. In general, the occurrence of specific adverse events was similar in both treatment groups. Pharyngitis was the most frequently reported adverse event in the ezetimibe-added-to-statin group (n = 3 [16.6%]). Vertigo was the most frequently reported adverse event in the statin group (n = 3 [18.8%]). There was 1 serious adverse event—an upper respiratory infection leading to hospitalization in the ezetimibe group, which was not considered drug-related by the investigator. There were no clinically meaningful differences in laboratory values between treatment groups at study end and no deaths occurred during the study.

**Table 2:** Summary of safety data

n (%)	**Ezetimibe** **(N = 42)**	**Statin** **(N = 41)**
Number of adverse events	18 (43)	16 (39)
≥1 adverse event	9 (21)	8 (20)
Serious adverse events	1 (2)	0 (0)
Serious drug-related adverse events	0 (0)	0 (0)
Deaths	0 (0)	0 (0)

**Table 3:** Frequency and percentage of adverse events in the subset of patients with at least one adverse event and serious adverse event by body system, and COSTART term

**Body System** **/COSTART**	**No. (%) of events**	
	**Ezetimibe (n = 18)**	**Statin (n = 16)**
**Body as a Whole**		
Fever	1 (5.6)	0
Headache	1 (5.6)	1 (6.3)
Pain back	1 (5.6)	0
Pain chest	1 (5.6)	0
**Cardiovascular System**		
Syncope	0	1 (6.3)
**Digestive System**		
Esophagitis	1 (5.6)	0
Nausea	1 (5.6)	1 (6.3)
Rectal discharge	1 (5.6)	0
**Metabolic and Nutritional Disorders**		
Diabetes mellitus	1 (5.6)	0
Gout	1 (5.6)	0
**Nervous System**		
Anxiety	1 (5.6)	0
Dizziness	2 (11.1)	1 (6.3)
Insomnia	0	1 (6.3)
Vertigo	0	3 (18.8)
**Respiratory System**		
Cough inc	0	1 (6.3)
Pharyngitis	3* (16.6)	1 (6.3)
Rhinitis	0	1 (6.3)
Sputum inc	0	1 (6.3)
Voice altered	0	1 (6.3)
**Skin and Appendages**		
Rash	1 (5.6)	0
**Special Senses**		
Cataract	1 (5.6)	0
Conjunctivitis	1 (5.6)	1 (6.3)
Deaf	0	1 (6.3)
Tinnitus	0	1 (6.3)

* One Serious Adverse Event.

## Discussion

After 8 weeks of treatment, ezetimibe added to statin resulted in significantly greater reductions in LDL-C and total cholesterol. Similar changes occurred in HDL-C and triglycerides in both treatment groups. The safety and tolerability profiles were generally similar for both treatments. These results are consistent with expectations for these treatment regimens in Caucasian patients and extend the results to Taiwanese patients.

The Hong Kong hospital audit study revealed that a considerable proportion of patients with dyslipidemia do not achieve LDL-C treatment targets on moderate dose statin [33]. Intensifying treatment is recommended. One option is to increase the dose of the statin until lipid targets are achieved or until the dose is not tolerated. If this strategy is not successful, adding a second therapy with a different mechanism of action to lower lipid levels (e.g., ezetimibe, fibrates, or niacin) may be the due course of action. In the Begin with the Real-world Patients of Non-goal-achieved Hypercholesterolemia in Taiwan through the Ezetimibe/Simvastatin Tablet (BRAVO) Study (N = 173 Taiwanese subjects across all cardiovascular risk categories), 91% of patients attained NCEP ATP III goals and experienced significant reductions in LDL-C, total cholesterol and triglyce-rides when treated with the combination ezetimibe/simvastatin [34]. Similarly, the results of this trial demonstrated that the addition of ezetimibe to a moderate dose of a statin is significantly more effective than doubling the dose of the statin for reducing LDL-C levels in Taiwanese patients. The subgroup analysis in diabetic and non-diabetic patients, although done in a limited number of subjects, is consistent with previous results in Western subjects showing that ezetimibe added to a statin reduces LDL-C more than doubling the statin dose in both diabetic and non-diabetic patients [35,36]. Larger trials in a broader Asian population base are warranted to provide appropriate statistical power and better interpretation of the results.

In addition, more patients achieved LDL-C <100 mg/dL with ezetimibe added to statin (58.6%) vs doubling the statin dose (41.2%). This difference was not significant; however, this may be due to the slightly (although not significantly) higher baseline LDL-C levels in the ezetimibe add-on group (143.4 mg/dL) compared with the statin group (129.8 mg/dL). It has been shown that higher baseline LDL-C was a significant negative predictor of LDL-C goal attainment, and this finding is consistent with the results reported here [33,37]. In addition, the small numbers of patients in this study provided limited power to detect statistically significant differences for the secondary endpoints.

Inter-individual variability in LDL-C lowering has been reported with both high-potency statins and ezetimibe [38,39]. It is notable that in this study adding ezetimibe reduced LDL-C in all but 3 individuals (26 out of 29 patients) and doubling statin reduced LDL-C in all but 12 individuals (22 out of 34 patients), but the amount of the decrease varied considerably between individuals. This finding is consistent with data from another study that showed variability in reductions in the fraction of cholesterol absorption with ezetimibe monotherapy, ezetimibe added to statin, and statin monotherapy [40]. The mechanism by which this variability occurs is poorly understood.

There is no test currently available that allows clinicians the ability to target lipid-lowering treatment accordingly. The only way to assess if a patient will attain treatment targets with statin monotherapy is through use of appropriate potency, dose and duration of statins. Clinical trial data support a complementary approach targeting the synthesis and the absorption of cholesterol to improve the lipid profile of patients who show a poor response to statin monotherapy [41-43]. More detailed analyses of patients categorized into specific statin groups may be of interest but would require a blinded study with a larger population.

In the present study, similar tolerability profiles were observed with both treatment regimens. The treatment group sizes were small and the study was not of sufficient duration to detect the presence of very rare adverse events. Despite these limitations, the tolerability results are consistent with expectations for these drugs at the doses given.

## Conclusions

In conclusion, this study demonstrated that ezetimibe added to ongoing statin therapy resulted in significantly greater lipid lowering compared with doubling the dose of statin in Taiwanese patients with hypercholesterolemia. Studies to assess clinical outcome benefit are ongoing.

## Abbreviations

ALT, Alanine aminotransferase; Apo B, Apolipoprotein B; AST, Aspartate aminotransferase; CHD, Coronary heart disease; CK, Creatine kinase; GGT, Gamma-glutamyl transpeptidase; HDL-C, High-density lipoprotein cholesterol; ITT, Intent-to-treat; LDL-C, Low-density lipoprotein cholesterol; NCEP ATP III, The National Cholesterol Education Program, Adult Treatment Panel III; TLCs, Therapeutic life changes; ULN, Upper limit of normal.

## Authors’ contributions

All authors meet the criteria for authorship stated in the Uniform Requirements for Manuscripts Submitted to Biomedical Journals. Specifically, **C-CY** collected or assembled data and interpreted the results; provided substantial suggestions for revision or critically reviewed subsequent iterations of the manuscript; reviewed and approved the final version of the manuscript, and provided study materials or patients. **W-TL** helped conceive, design or plan the study and collected or assembled the data; provided substantial suggestions for revision or critically reviewed subsequent iterations of the manuscript; approved the final version of the manuscript, and provided study materials or patients. **K-CS** helped conceive, design or plan the study; performed or supervised analyses and interpreted the results and collected or assembled the data; provided substantial suggestions for revision or critically reviewed subsequent iterations of the manuscript; reviewed and approved the final version of the manuscript and provided study materials or patients and administrative, technical or logistic support. **T-HL** collected or assembled the data; provided substantial suggestions for revision or critically reviewed subsequent iterations of the manuscript; reviewed and approved the final version of the manuscript and provided study materials or patients. **C-HL** collected or assembled the data; provided substantial suggestions for revision or critically reviewed subsequent iterations of the manuscript; reviewed and approved the final version of the manuscript and provided study materials or patients. **H-JL** interpreted the results; provided substantial suggestions for revision or critically reviewed subsequent iterations of the manuscript; and reviewed and approved the final version of the manuscript **MH** interpreted the results; wrote sections of the initial draft; provided substantial suggestions for revision or critically reviewed subsequent iterations of the manuscript; and reviewed and approved the final version of the manuscript. **J-JH** interpreted the results; wrote sections of the initial draft; provided substantial suggestions for revision or critically reviewed subsequent iterations of the manuscript; and reviewed and approved the final version of the manuscript. All authors read and approved the final manuscript.

## Competing interests

This study was funded by MSD Taiwan.

Drs. Chih-Chieh Yu, Wen-Ter Lai, Tsung-Hsien Lin and Juey-Jen Hwang report no conflicts of interest regarding this manuscript. Dr. Kuang-Chung Shih and Dr. Chieh-Hua Lu received a grant and a consulting fee or honorarium from the study sponsor for the conduct of this study. Dr. Hung-Jen Lai is an employee of MSD Taiwan and may hold stock or stock options in the company. Dr. Mary E. Hanson is an employee of Merck Sharp & Dohme Corp., a subsidiary of Merck & Co., Whitehouse Station NJ, and may hold stock or stock options in the company.
